# A cost-benefit framework for prosocial motivation—Advantages and challenges

**DOI:** 10.3389/fpsyt.2023.1170150

**Published:** 2023-03-24

**Authors:** Luis Sebastian Contreras-Huerta

**Affiliations:** ^1^Center for Social and Cognitive Neuroscience (CSCN), School of Psychology, Universidad Adolfo Ibáñez, Viña del Mar, Chile; ^2^Center for Human Brain Health, School of Psychology, University of Birmingham, Birmingham, United Kingdom

**Keywords:** prosocial behavior, cost-benefit decision-making, individual differences, global challenges, psychiatric traits

## Prosocial motivation as cost-benefit decisions

In everyday life, people help others, from assisting colleagues with a task, to holding doors for strangers. These types of actions have been called prosocial behaviors—actions that benefit others (1). These behaviors are found not only in humans but also in a wide range of species where individual actions increase the fitness of a group, facilitating social cohesion (2–4). In humans these behaviors are very versatile and vary between contexts and individuals. Characteristics of the beneficiary (e.g., family vs. strangers), of the agent (e.g., personality traits), and the context (e.g., type of benefit), all influence how willing people are to help others (4–9). Understanding how these characteristics modulate willingness to help is important to uncover why there is variability in prosocial behavior.

Here, I propose that a group of prosocial behaviors could be conceptualized as goal-directed actions (10)—actions that benefit others at the agent's own cost. The agent needs to be incentivized by the social benefit to overcome the cost and perform the action. Thus, in this group of actions, the material and direct benefit of the action is not received by the agents themselves but by others. The probability of a prosocial action to occur will depend then, among other factors, on how costly and how beneficial the action is—e.g., high benefits for low costs would have a high probability for the prosocial action to occur and vice versa. Crucially, this cost-benefit process is highly subjective—e.g., some people could be highly sensitive to others' welfare, and be prosocial for little benefit regardless of the cost, while others could be very sensitive to the cost, refraining to act even if the cost is low, regardless of the social benefit.

This framework can be conceptually and methodologically powerful when addressing prosocial motivation. First, it can account for different types of behaviors that, in appearance, might not seem necessarily related. For instance, effortful prosocial actions, where effort costs need to be overcome to benefit others; and harm aversion, where costs are incurred to decrease others' suffering, have been linked empirically under a cost-benefit framework (11). Thus, costs (e.g., effort, time, money, distress) and benefits (e.g., money, avoiding harm, food, emotional support) can vary across contexts, but the decisional process might share similar principles. Secondly, using computational models, this framework allows us to understand the mechanisms underlying prosocial behaviors across different contexts, while capturing the idiosyncratic cost-benefit evaluations performed by individuals (10, 12, 13). Finally, costs and benefits can be manipulated and measured separately in experimental paradigms, accounting for individual variability in the sensitivities that people have to each element of the decision (10). Considering all of the above, in the following I will describe how a cost-benefit framework could be useful to address inter-individual and inter-contextual variability in prosocial behavior.

## Inter-individual variability—Psychiatric and affective traits

Prosocial behavior is known for impacting social relationships, life satisfaction, economic success as well as mental and physical health (14–16), and its alteration, together with socio-affective disruption, have been linked to personality, depressive and anxiety disorders (6, 17–24). The propensity to act prosocially may therefore vary with psychiatric traits.

Crucially, new perspectives in psychiatry have proposed disorders as one extreme of a continuum where different traits are present to certain degrees (25, 26). Thus, healthy and neurodiverse populations vary only in the magnitude and combination of strength of subclinical traits. Many subclinical traits have been associated with prosocial disruption. For instance, psychopathy, alexithymia and apathy have all been linked to impaired prosocial behavior and social cognition (9, 18, 27–31). Other traits, disrupted across psychiatric disorders such as empathy and interoception (20, 32–35), have been linked to facilitating prosocial actions (36–39). Importantly, all these subclinical traits are not independent from each other (30, 40–42). Thus, how people evaluate the costs and benefits of being prosocial, or some broader impact on mood or willingness to act in general, is unclear.

A cost-benefit framework can illuminate how these different variables interact and influence people's prosocial decision-making process. In an online study (11), we measured willingness to help in two different contexts, one where the benefit was monetary and the cost was effort (prosocial effort), and another where the cost was financial, and the benefit was alleviating pain (harm aversion). Behavior in both tasks were correlated, supporting the idea of cost-benefit evaluations across contexts. Crucially, we also measured affective and psychiatric traits to identify which constellations of these traits most strongly predicted prosociality. We found that general subclinical traits associated with affective reactivity, such as high empathy but low alexythimia and emotional apathy, more strongly explained prosocial variability in these two contexts, rather than psychiatric traits such as depression and anxiety. These results suggest that people who are highly sensitive to their own and others' emotions are more likely to behave prosocially. Consistently, in a further lab-based experiment, we found that interoception was associated with prosocial behavior, but specifically related to sensitivity to others' benefits (43). Thus, people who were more aware of their bodily signals were more incentivized by the reward that others received, working more to obtain them. These results support the idea of high sensitivity to own and others' states as an important motor for cost-benefit prosociality. Future directions should disentangle further the relationship between psychiatric disorders and the specific elements in a prosocial decision using computational models, such as drift-diffusion models. These models have the potential to unveil which psychiatric or subclinical traits are linked to what specific aspect of the cost-benefit decisional process (44–46).

This cost-benefit, transdiagnostic approach can also contribute to modern psychiatry research. Advances in diagnosis and treatment of psychiatric symptoms have been improved from evidence-based research, such as the Research Domain Criteria (RDoC) initiative, which unifies biological and cognitive markers across specific domains in psychiatry (47). Likewise, computational psychiatry has revealed cognitive mechanisms underlying psychiatric traits, identifying protective factors and improving diagnostic tools (48, 49). A cost-benefit prosocial framework could help to understand social disruption in the psychiatric-healthy spectrum, illuminating the mechanisms of prosocial behavior and characterize individual variability. Progress in understanding the computational underpinnings of cost-benefit prosocial decisions has already been made, identifying differences between people on how specific elements of the decisional process influence prosocial behavior (8, 9, 45). These mechanisms have been linked to neural patterns of activity associated with individual variability in willingness to help others (10, 45, 50–52). Thus, neurocomputational models of cost-benefit prosocial decisions might be a powerful tool to capture transdiagnostic psychiatric profiles linked to social disruption, similar to previous computational psychiatry advances addressing other cognitive domains (53, 54).

## Inter-contextual variability—Major global challenges

Prosocial behavior is essential for addressing major global challenges of the 21st century, such as infectious diseases and climate change (55–58). However, it has been shown that people are averse to engaging in pro-environmental behavior (59), and a considerable amount of people did not engage in behaviors that prevented the spread of COVID-19 (60).

These challenges can be seen under a cost-benefit prosocial framework. During the COVID-19 pandemic for instance, using masks or isolating from others not only affected the agents themselves but also the health of other people around them (58). Thus, these behaviors can be seen as harm aversion decisions, where the personal cost can benefit others by preventing their harm. Likewise, pro-environmental behavior can be considered as prosocial since it requires making decisions with outcomes for other people (56). Many pro-environmental behaviors, such as recycling or campaigning for more environmental actions, require putting in effort to benefit society. Thus, they involve a cost-benefit decision, where effort (or other) costs are incurred toward the welfare of people in general. Indeed, studies have shown that when the personal cost is lower and the environmental benefit is higher, pro-environmental behavior increases (61–64), and it is better predicted by pro-environmental attitudes (61). Therefore, understanding people's willingness to act in a way that helps toward global challenges can also be considered as linked to cost-benefit decision-making.

From a cost-benefit perspective, actions that alleviate these global challenges could bring material and direct benefits to the agents who perform them. However, this is not necessarily the case. For instance, people might not see risk for themselves during an outbreak of an infectious disease due to demographic and health characteristics (65, 66). Furthermore, generational gaps can exist in attitudes toward climate change, as the most severe consequences of global warming might not be seen for older adults (67, 68). Thus, the cost of prosocial actions within these global challenges must be still incurred by the agent, but the benefits are received by others seen as more vulnerable to their consequences.

Even the benefit in such global challenges present specific characteristics—e.g., they are uncertain in their target (who will be benefitted specifically?), time (when will they be benefitted?), and impact. Thus, this reframing may be useful, as costs and benefits can be dissociated and manipulated in such a way that different scenarios can be emulated, and the mechanisms and the variables that affect prosocial decisions in specific contexts can be compared. Furthermore, by reframing these challenges as cost-benefit decision-making problems, it may be possible to precisely measure whether people are actually willing to act prosocially to meet these global challenges, rather than increasing their desire to help without actually changing behavior. Indeed, recently we found that people are highly hypocritical in a cost-benefit, harm aversion context, judging others' misconduct harshly even when they themselves have committed the same transgressions (69). This hypocritical behavior was associated with behavioral and neural markers of guilt, suggesting that people might desire a prosocial goal, but feel frustrated when their will fails. Given that in these global challenges a high degree of hypocritical blame occurs (e.g., blaming others for transgressing COVID-19 regulations while throwing crowded parties themselves), this cost-benefit approach could unveil the mechanisms underlying these behaviors, potentially facilitating prosociality.

A different methodological approach also would be beneficial to study behavior in these global challenges. For instance, most studies on climate change have relied on self-reports with no real consequences for the environment (63, 70), and on small samples of western, largely student, populations who might not represent behaviors across the globe (71). With online settings, these barriers can be more easily broken, and a more global sample reached. This approach has already been used during the COVID-19 pandemic in a prosocial context. In data collected online from countries across five continents, a study found that distancing behavior during COVID-19 pandemic was correlated with hypothetical charity donations (72). This supports the notion that different cost-benefit prosocial decisions can share variance. Interestingly, this study also examined links with another global challenge, i.e., aging, which might be linked to differences in cost-benefit evaluations (73). A cost-benefit framework could allow us to comprehend contextual variability, identifying those factors that can modulate prosociality within global challenges. Global, online approaches are crucial for this, and can be expanded to other global challenges such as climate change (e.g., https://manylabsclimate.wordpress.com/).

## Limitations and challenges for prosociality research

Here, I have presented a cost-benefit framework for prosocial motivation where an agent must incur a cost to benefit someone else. This framework could be useful to disentangle the mechanisms underpinning variability in prosocial decisions across individuals and contexts. Identifying variables that could foment prosociality in the population might act as a protective factor not only for mental and physical health, but also to address global challenges of our time.

Despite the advantages that this framework might have, an obvious limitation is that it does not necessarily account for all types of prosocial behaviors. For instance, there is a group of prosocial behaviors that can bring some material benefits for the agent themselves, such as cooperation and reciprocity (4). Likewise, factors other than cost-benefit evaluations can modulate prosociality, such as social norms and moral values (74, 75). Furthermore, costs and benefits can change their value depending on the opportunity-costs of the environment, something that some studies have started to look at in prosocial contexts (76). In addition, in some contexts, costs could be rewarding in their own right (77), even if the benefit is for someone else (78). However, more than a direct criticism, these challenges add complexity to this framework, inviting to expand it to other levels within a cost-benefit decisional process.

I have argued that a cost-benefit framework for prosocial behavior could be useful to reduce global threats. Nevertheless, how to translate discoveries in behavioral sciences to the public domain has proved challenging (79). There are some signs however that this could change in the following years. In the middle of COVID-19 pandemic, a seminal article summarized relevant findings in our field, suggesting policies that could help to face the emergency (80). Even though some criticized this piece (81), a recent preprint concluded that around 85% of the suggestions were supported by evidence produced during the pandemic (82). Innovatively, they also assessed the quality of empirical evidence considering impact in policy making. Given previous work on decision-making research informing policy making (83), a prosocial cost-benefit framework could be one of the topics that could benefit society if applied in the real world. This not only will help to combat global challenges, but will also prove whether the knowledge we have accumulated in our discipline makes sense in the wild (84). Diversifying our samples and creating knowledge from countries in the Big South might be crucial to achieve these goals (85, 86). Understanding prosocial behavior could potentially have a positive impact on the lives of many people across the world, and this cost-benefit framework might be an important contributor to this end.

## Author contributions

The author confirms being the sole contributor of this work and has approved it for publication.

## References

[1] Schroeder DA Graziano WG. The Oxford Handbook of Prosocial Behavior. (2015) 10.1093/oxfordhb/9780195399813.013.32

[2] de Waal FBM. Putting the altruism back into altruism: the evolution of empathy. Annu Rev Psychol. (2008) 59:279–300. 10.1146/annurev.psych.59.103006.093625 17550343

[3] Hamilton WD. The evolution of altruistic behavior. Am Nat. (1963) 97:354–6. 10.1086/497114

[4] Kurzban R Burton-Chellew MN West SA. The evolution of altruism in humans. Annu Rev Psychol. (2015) 66:575–99. 10.1146/annurev-psych-010814-015355 25061670

[5] White BA. Who cares when nobody is watching? Psychopathic traits and empathy in prosocial behaviors. Pers Individ Dif. (2014) 56:116–21. 10.1016/j.paid.2013.08.033

[6] Robson SE Repetto L Gountouna V-E Nicodemus KK. A review of neuroeconomic gameplay in psychiatric disorders. Mol Psychiatry. (2019) 1:5. 10.1038/s41380-019-0405-5 31040383 PMC6906183

[7] Bernhard H Fischbacher U Fehr E. Parochial altruism in humans. Nature. (2006) 442:912–5. 10.1038/nature04981 16929297

[8] Crockett MJ Kurth-Nelson Z Siegel JZ Dayan P Dolan RJ. Harm to others outweighs harm to self in moral decision making. Proc Natl Acad Sci. (2014) 111:173201–17325. 10.1073/pnas.1408988111 25404350 PMC4260587

[9] Lockwood PL Hamonet M Zhang SH Ratnavel A Salmony FU Husain M. Prosocial apathy for helping others when effort is required. Nat Hum Behav. (2017) 1:0131. 10.1038/s41562-017-0131 28819649 PMC5555390

[10] Contreras-Huerta LS Pisauro MA Apps MAJ. Effort shapes social cognition and behaviour: A neuro-cognitive framework. Neurosci Biobehav Rev. (2020) 118:426–39. 10.1016/j.neubiorev.2020.08.003 32818580

[11] Contreras-Huerta LS Lockwood PL Bird G Apps MAJ Crockett MJ. Prosocial behavior is associated with transdiagnostic markers of affective sensitivity in multiple domains. Emotion. (2020) 22:820. 10.1037/emo0000813 32718171 PMC9301775

[12] Crockett MJ. How formal models can illuminate mechanisms of moral judgment and decision making. Curr Dir Psychol Sci. (2016) 25:85–90. 10.1177/0963721415624012

[13] Lockwood PL Klein-Flügge MC. Computational modelling of social cognition and behaviour—a reinforcement learning primer. Soc Cogn Affect Neurosci. (2020) 3:1–11. 10.1093/scan/nsaa040 32232358 PMC8343561

[14] Brown SL Brown RM. Connecting prosocial behavior to improved physical health: contributions from the neurobiology of parenting. Neurosci Biobehav Rev. (2015) 55:1–17. 10.1016/j.neubiorev.2015.04.004 25907371

[15] Post SG. Altruism, happiness, and health: it's good to be good. Int J Behav Med. (2005) 12:66–77. 10.1207/s15327558ijbm1202_4 15901215

[16] Stern GR. Altruism: giving for mental wellbeing. J Am Psychiatr Nurses Assoc. (2019) 25:314–5. 10.1177/1078390319857080 31262244

[17] American Psychiatric Association. Diagnostic and Statistical Manual of Mental Disorders (DSM-5^®^). Washington, DC: American Psychiatric Pub (2013). 10.1176/appi.books.9780890425596

[18] James R Blair R. The neurobiology of psychopathic traits in youths. Nat Rev Neurosci. (2013) 14:786–99. 10.1038/nrn3577 24105343 PMC4418507

[19] Blair RJR. The amygdala and ventromedial prefrontal cortex in morality and psychopathy. Trends Cogn Sci. (2007) 11:387–92. 10.1016/j.tics.2007.07.003 17707682

[20] Herpertz SC Bertsch K. The social-cognitive basis of personality disorders. Curr Opin Psychiatry. (2014) 27:73–7. 10.1097/YCO.0000000000000026 24270477

[21] Mier D Lis S Esslinger C Sauer C Hagenhoff M Ulferts J. Neuronal correlates of social cognition in borderline personality disorder. Soc Cogn Affect Neurosci. (2013) 8:531–7. 10.1093/scan/nss028 22362841 PMC3682436

[22] Rilling JK Glenn AL Jairam MR Pagnoni G Goldsmith DR Elfenbein HA. Neural correlates of social cooperation and non-cooperation as a function of psychopathy. Biol Psychiatry. (2007) 61:1260–71. 10.1016/j.biopsych.2006.07.021 17046722

[23] Unoka Z Seres I Áspán N Bódi N Kéri S. Trust game reveals restricted interpersonal transactions in patients with borderline personality disorder. J Pers Disord. (2009) 23:399–409. 10.1521/pedi.2009.23.4.399 19663659

[24] Yu R Geddes JR Fazel S. Personality disorders, violence, and antisocial behavior: a systematic review and meta-regression analysis. J Pers Disord. (2012) 26:775–92. 10.1521/pedi.2012.26.5.775 23013345

[25] McGorry P Nelson B. Why we need a transdiagnostic staging approach to emerging psychopathology, early diagnosis, and treatment. JAMA Psychiatry. (2016) 73:191–2. 10.1001/jamapsychiatry.2015.2868 26765254

[26] Braun U. A Network perspective on the search for common transdiagnostic brain mechanisms. Biol Psychiatry. (2018) 84:e47–8. 10.1016/j.biopsych.2018.07.017 30165953

[27] Demers LA Koven NS. The relation of alexithymic traits to affective theory of mind. Am J Psychol. (2015) 128:31–42. 10.5406/amerjpsyc.128.1.0031 26219172

[28] FeldmanHall O Dalgleish T Mobbs D. Alexithymia decreases altruism in real social decisions. Cortex. (2013) 49:899–904. 10.1016/j.cortex.2012.10.015 23245426

[29] Grynberg D Luminet O Corneille O Grèzes J Berthoz S. Alexithymia in the interpersonal domain: a general deficit of empathy? Pers Individ Dif. (2010) 49:845–50. 10.1016/j.paid.2010.07.01333458324

[30] Valdespino A Antezana L Ghane M Richey JA. Alexithymia as a transdiagnostic precursor to empathy abnormalities: the functional role of the insula. Front Psychol. (2017) 8:1–7. 10.3389/fpsyg.2017.02234 29312079 PMC5742870

[31] Pauli R Lockwood PL. The computational psychiatry of antisocial behaviour and psychopathy. Neurosci Biobehav Rev. (2022) 3:104995. 10.31234/osf.io/mqbvu 36535376

[32] Bonaz B Lane RD Oshinsky ML Kenny PJ Sinha R Mayer EA. Diseases, disorders, and comorbidities of interoception. Trends Neurosci. (2021) 44:39–51. 10.1016/j.tins.2020.09.009 33378656

[33] Khalsa SS Adolphs R Cameron OG Critchley HD Davenport PW Feinstein JS. Interoception and mental health: a roadmap. Biol Psychiatry Cogn Neurosci Neuroimag. (2018) 3:501–13. 10.1016/j.bpsc.2017.12.004 29884281 PMC6054486

[34] Bird G Viding E. The self to other model of empathy: providing a new framework for understanding empathy impairments in psychopathy, autism, and alexithymia. Neurosci Biobehav Rev. (2014) 47:520–32. 10.1016/j.neubiorev.2014.09.021 25454356

[35] New AS aan het Rot M Ripoll LH Perez-Rodriguez MM Lazarus S Zipursky E. Empathy and alexithymia in borderline personality disorder: clinical and laboratory measures. J Pers Disord. (2012) 26:660–75. 10.1521/pedi.2012.26.5.660 23013336

[36] Decety J Bartal IB-A Uzefovsky F Knafo-Noam A. Empathy as a driver of prosocial behaviour: highly conserved neurobehavioural mechanisms across species. Philos Trans R Soc B Biol Sci. (2016) 371:20150077. 10.1098/rstb.2015.0077 26644596 PMC4685523

[37] FeldmanHall O Dalgleish T Evans D Mobbs D. Empathic concern drives costly altruism. Neuroimage. (2015) 105:347–56. 10.1016/j.neuroimage.2014.10.043 25462694 PMC4275572

[38] Morelli SA Rameson LT Lieberman MD. The neural components of empathy: Predicting daily prosocial behavior. Soc Cogn Affect Neurosci. (2014) 9:39–47. 10.1093/scan/nss088 22887480 PMC3871722

[39] Piech RM Strelchuk D Knights J Hjälmheden J V Olofsson JK Aspell JE. People with higher interoceptive sensitivity are more altruistic, but improving interoception does not increase altruism. Sci Rep. (2017) 7:1–5. 10.1038/s41598-017-14318-8 29142226 PMC5688159

[40] Lockwood PL Ang YS Husain M Crockett MJ. Individual differences in empathy are associated with apathy-motivation. Sci Rep. (2017) 7:17293. 10.1038/s41598-017-17415-w 29229968 PMC5725487

[41] Brewer R Cook R Bird G. Alexithymia: a general deficit of interoception. R Soc open Sci. (2016) 3:150664. 10.1098/rsos.150664 27853532 PMC5098957

[42] Lischke A Weippert M Mau-Moeller A Jacksteit R Pahnke R. Interoceptive accuracy is associated with emotional contagion in a valence- and sex-dependent manner. Soc Neurosci. (2020) 15:227–33. 10.1080/17470919.2019.1690573 31693866

[43] Contreras-Huerta LS Coll M-P Bird G Yu H Prosser A Lockwood PL. Neural representations of vicarious rewards are linked to interoception and prosocial behaviour. Neuroimage. (2023) 3:119881. 10.1016/j.neuroimage.2023.119881 36702212

[44] Chen F Krajbich I. Biased sequential sampling underlies the effects of time pressure and delay in social decision making. Nat Commun. (2018) 9:3557. 10.1038/s41467-018-05994-9 30177719 PMC6120923

[45] Hutcherson CA Bushong B Rangel A. A neurocomputational model of altruistic choice and its implications. Neuron. (2015) 87:451–62. 10.1016/j.neuron.2015.06.031 26182424 PMC4947370

[46] Saulin A Horn U Lotze M Kaiser J Hein G. The neural computation of human prosocial choices in complex motivational states. Neuroimage. (2022) 247:118827. 10.1016/j.neuroimage.2021.118827 34923133

[47] Insel T Cuthbert B Garvey M Heinssen R Pine D Quinn K. Research domain criteria (RDoC): toward a new classification framework for research on mental disorders. Am J Psychiatry Online. (2010) 25:748–51. 10.1176/appi.ajp.2010.09091379 20595427

[48] Huys QJM Maia T V Frank MJ. Computational psychiatry as a bridge from neuroscience to clinical applications. Nat Neurosci. (2016) 19:404–13. 10.1038/nn.4238 26906507 PMC5443409

[49] Wang XJ Krystal JH. Computational psychiatry. Neuron. (2014) 84:638–54. 10.1016/j.neuron.2014.10.018 25442941 PMC4255477

[50] Hu J Hu Y Li Y Zhou X. Computational and neurobiological substrates of cost-benefit integration in altruistic helping decision. J Neurosci. (2021) 41:3545–61. 10.1523/JNEUROSCI.1939-20.2021 33674417 PMC8051690

[51] Crockett MJ Siegel JZ Kurth-Nelson Z Dayan P Dolan RJ. Moral transgressions corrupt neural representations of value. Nat Neurosci. (2017) 20:879–85. 10.1038/nn.4557 28459442 PMC5462090

[52] Lockwood PL Wittmann MK Nili H Matsumoto-Ryan M Abdurahman A Cutler J. Distinct neural representations for prosocial and self-benefiting effort. Curr Biol. (2022) 32:4172–85. 10.1016/j.cub.2022.08.010 36029773 PMC9616728

[53] Gillan CM Kosinski M Whelan R Phelps EA Daw ND. Characterizing a psychiatric symptom dimension related to deficits in goal- directed control. Elife. (2016) 5:1–24. 10.7554/eLife.11305 26928075 PMC4786435

[54] Rouault M Seow T Gillan CM Fleming SM. Psychiatric symptom dimensions are associated with dissociable shifts in metacognition but not task performance. Biol Psychiatry. (2018) 84:443–51. 10.1016/j.biopsych.2017.12.017 29458997 PMC6117452

[55] Klein SA Nockur L Reese G. Prosociality from the perspective of environmental psychology. Curr Opin Psychol. (2022) 44:182–7. 10.1016/j.copsyc.2021.09.001 34695642

[56] Nolan JM Schultz PW. Prosocial behavior and environmental action. In:Schroeder DA Graziano WG editors. The Oxford Handbook of Prosocial Behavior. Oxford University Press (2015). p. 626–52.

[57] Böhm R Betsch C. Prosocial vaccination. Curr Opin Psychol. (2022) 43:307–11. 10.1016/j.copsyc.2021.08.010 34517200

[58] Jordan JJ Yoeli E Rand DG. Don't get it or don't spread it: Comparing self-interested versus prosocial motivations for COVID-19 prevention behaviors. Sci Rep. (2021) 11:20222. 10.1038/s41598-021-97617-5 34642341 PMC8511002

[59] Markowitz EM Shariff AF. Climate change and moral judgement. Nat Clim Chang. (2012) 2:243–7. 10.1038/nclimate1378

[60] Harris LC. Breaking lockdown during lockdown: a neutralization theory evaluation of misbehavior during the Covid 19 pandemic. Deviant Behav. (2022) 43:765–79. 10.1080/01639625.2020.1863756

[61] Wyss AM Knoch D Berger S. When and how pro-environmental attitudes turn into behavior: the role of costs, benefits, and self-control. J Environ Psychol. (2022) 79:101748. 10.1016/j.jenvp.2021.101748

[62] Berger S Kilchenmann A Lenz O Ockenfels A Schlöder F Wyss AM. Large but diminishing effects of climate action nudges under rising costs. Nat Hum Behav. (2022) 6:1381–5. 10.1038/s41562-022-01379-7 35739251

[63] Lange F Steinke A Dewitte S. the pro-environmental behavior task: a laboratory measure of actual pro-environmental behavior. J Environ Psychol. (2018) 56:46–54. 10.1016/j.jenvp.2018.02.007

[64] Lange F Dewitte S. The Work for environmental protection task: a consequential web-based procedure for studying pro-environmental behavior. Behav Res Methods. (2022) 54:133–45. 10.3758/s13428-021-01617-2 34109560

[65] Nino M Harris C Drawve G Fitzpatrick KM. Race and ethnicity, gender, and age on perceived threats and fear of COVID-19: evidence from two national data sources. SSM-population Heal. (2021) 13:100717. 10.1016/j.ssmph.2020.100717 33344747 PMC7733547

[66] Sattler S Taflinger S Ernst A Hasselhorn F. A Moderated mediation model explaining the relationship between risk-group membership, threat perception, knowledge, and adherence to COVID-19 behavioral measures. Front Public Heal. (2022) 10:2368. 10.3389/fpubh.2022.842368 35664099 PMC9160797

[67] Rothermich K Johnson EK Griffith RM Beingolea MM. The influence of personality traits on attitudes towards climate change—An exploratory study. Pers Individ Dif. (2021) 168:110304. 10.1016/j.paid.2020.110304

[68] Hamilton LC Hartter J Bell E. Generation gaps in US public opinion on renewable energy and climate change. PLoS ONE. (2019) 14:e0217608. 10.1371/journal.pone.0217608 31291254 PMC6619988

[69] Yu H Contreras-Huerta LS Prosser AMB Apps MAJ Hofmann W Sinnott-Armstrong W. Neural and cognitive signatures of guilt predict hypocritical blame. Psychol Sci. (2022) 33:1909–27. 10.1177/09567976221122765 36201792

[70] Lange F Dewitte S. Measuring pro-environmental behavior: review and recommendations. J Environ Psychol. (2019) 63:92–100. 10.1016/j.jenvp.2019.04.009

[71] Tam K-P Milfont TL. Towards cross-cultural environmental psychology: a state-of-the-art review and recommendations. J Environ Psychol. (2020) 71:101474. 10.1016/j.jenvp.2020.101474

[72] Cutler J Nitschke JP Lamm C Lockwood PL. Older adults across the globe exhibit increased prosocial behavior but also greater in-group preferences. Nat Aging. (2021) 1:880–8. 10.1038/s43587-021-00118-3 PMC10154238 37118329

[73] Lockwood PL Abdurahman A Gabay AS Drew D Tamm M Husain M. Aging increases prosocial motivation for effort. Psychol Sci. (2021) 32:668–81. 10.1177/0956797620975781 33860711 PMC7611497

[74] Daniel Batson C Ahmad N Tsang JA. Four motives for community involvement. J Soc Issues. (2002) 58:429–45. 10.1111/1540-4560.00269

[75] Crockett MJ. Models of morality. Trends Cogn Sci. (2013) 17:363–6. 10.1016/j.tics.2013.06.005 23845564 PMC3925799

[76] Contreras-Huerta LS Pisauro A Kuechenhoff S Gekiere A Le Heron C Lockwood P. A reward self-bias leads to more optimal foraging for ourselves than others. PsyArXiv. (2022) 10.31234/osf.io/8r45z PMC11538449 39500761

[77] Inzlicht M Shenhav A Olivola CY. The effort paradox: effort is both costly and valued. Trends Cogn Sci. (2018) 5:2. 10.31234/osf.io/b5a2m 29477776 PMC6172040

[78] Olivola CY Shafir E. The martyrdom effect: when pain and effort increase prosocial contributions. J Behav Decis Mak. (2013) 26:91–105. 10.1002/bdm.767 23559692 PMC3613749

[79] Oliver K Lorenc T Innvær S. New directions in evidence-based policy research: a critical analysis of the literature. Heal Res policy Syst. (2014) 12:1–11. 10.1186/1478-4505-12-34 25023520 PMC4107868

[80] Bavel JJ Baicker K Boggio PS Capraro V Cichocka A Cikara M. Using social and behavioural science to support COVID-19 pandemic response. Nat Hum Behav. (2020) 4:460–71. 10.1038/s41562-020-0884-z 32355299

[81] IJzerman H Lewis NA Jr Przybylski AK Weinstein N DeBruine L Ritchie SJ. Use caution when applying behavioural science to policy. Nat Hum Behav. (2020) 4:1092–4. 10.1038/s41562-020-00990-w 33037396

[82] Ruggeri K Stock F Haslam SA Capraro V Boggio P Ellemers N. Evaluating expectations from social and behavioral science about COVID-19 and lessons for the next pandemic. PsyArXiv. (2022). 10.31234/osf.io/58udn

[83] Sunstein CR. The council of psychological advisers. Annu Rev Psychol. (2016) 67:713–37. 10.1146/annurev-psych-081914-124745 26393867

[84] Ibanez A. The mind's golden cage and cognition in the wild. Trends Cogn Sci. (2022) 3:8. 10.1016/j.tics.2022.07.008 36243670 PMC11197079

[85] Henrich J Heine SJ Norenzayan A. Most people are not WEIRD. Nature. (2010) 466:29. 10.1038/466029a 20595995

[86] Duran-Aniotz C Sanhueza J Grinberg LT Slachevsky A Valcour V Robertson I. The Latin American brain health institute, a regional initiative to reduce the scale and impact of dementia. Wiley Online Library. (2022) 5:12710. 10.1002/alz.12710 35708193 PMC9482938

